# Mediterranean Wild Edible Plants: Weeds or “New Functional Crops”?

**DOI:** 10.3390/molecules23092299

**Published:** 2018-09-08

**Authors:** Costanza Ceccanti, Marco Landi, Stefano Benvenuti, Alberto Pardossi, Lucia Guidi

**Affiliations:** 1 Department of Agriculture, Food & Environment, University of Pisa, Via del Borghetto, 80-56124 Pisa, Italy; costanza.ceccanti1811@gmail.com (C.C.); marco.landi@agr.unipi.it (M.L.); stefano.benvenuti@unipi.it (S.B.); alberto.pardossi@unipi.it (A.P.); 2 Interdepartmental Research Center Nutrafood-Nutraceuticals and Food for Health, University of Pisa, Via del Borghetto, 80-56124 Pisa, Italy

**Keywords:** functional food, hydroponic system, Mediterranean diet, oxalic acid, phytochemicals, toxic compound, wild species

## Abstract

The Mediterranean basin is a biodiversity hotspot of wild edible species, and their therapeutic and culinary uses have long been documented. Owing to the growing demand for wild edible species, there are increasing concerns about the safety, standardization, quality, and availability of products derived from these species collected in the wild. An efficient cultivation method for the species having promising nutraceutical values is highly desirable. In this backdrop, a hydroponic system could be considered as a reproducible and efficient agronomic practice to maximize yield, and also to selectively stimulate the biosynthesis of targeted metabolites. The aim of this report is to review the phytochemical and toxic compounds of some potentially interesting Mediterranean wild edible species. Herein, after a deep analysis of the literature, information on the main bioactive compounds, and some possibly toxic molecules, from fifteen wild edible species have been compiled. The traditional recipes prepared with these species are also listed. In addition, preliminary data about the performance of some selected species are also reported. In particular, germination tests performed on six selected species revealed that there are differences among the species, but not with crop species. “Domestication” of wild species seems a promising approach for exploiting these “new functional foods”.

## 1. Introduction

Since ancient times, wild plants have widely been used in traditional Mediterranean culture, and the link between wild plants and human life is a prominent feature. Wild plants are known to be used in ancient cultures for different purposes, such as food, medicines, production of goods (for example clothes), and magic and religious rituals. In particular, the use of wild edible plants in Europe has been mainly linked to periods of famine, therefore these herbs are called “famine food” [1]. Through the years, the use of these plants in traditional recipes of the Mediterranean diet has continuously increased, and in parallel, people have discovered their medicinal properties [2]. Today, the renewed interest in wild edible plants, and knowledge of the healthy role of phytochemical compounds, makes it possible to define them as “new functional foods”. On the other hand, strong concern about safety, yield, and the phytochemical profiles of these species, makes it crucially important to establish a large-scale methodology of cultivation of the most promising species, in terms of both nutraceutical value and profitability. The hydroponic system represents a reproducible and efficient agronomic practice to maximize not only yield, but also to selectively stimulate the biosynthesis of targeted metabolites [3,4]. Another important aspect worth further analysis is the high variability in the percentage and mean germination time of wild edible species [5,6].

## 2. Wild Edible Plants in the Mediterranean Basin 

The Mediterranean basin is characterized by a massive abundance of wild edible species. Of the selected fifteen wild species appearing to be the most promising for cultivation, the most representative compounds are detailed in Table 1. A plethora of bioactive compounds with medicinal and nutraceutical properties have been isolated from these species. Of them, silenan SV from *Sinapis arvensis* L. with immunomodulatory activity [7], and alliin in *Allium ampeloprasum* L. with powerful antioxidant activity [1], are well-known examples. Wild species are constitutively rich in secondary metabolites with antioxidant and healthy properties, and for these reasons could be represented as a new source of functional food. On the other hand, many of these properties were already known, even though not scientifically proven.

There is a difference between developing and industrialized countries in their habits of consumption of wild species. In developing nations, many edible wild plants are used as a source of food because the domesticated crop yield is not sufficient, whereas in most industrialized countries food supply is not a problem, thus wild plants are used to diversify a monotonous diet. Today, the concept of food in developed countries is profoundly modified. Indeed, consumers are no longer interested only in the supply of basic nutrients, they also demand the contribution of nutraceutical compounds.

The Mediterranean diet is rich in traditional dishes with wild edible species cooked in different ways, such as soups, pies, mixtures, boiled vegetables, and ravioli. According to popular tradition, some culinary uses of the species are reported in Table 2.

## 3. Toxicity of Wild Edible Plants 

A high accumulation of nitrites, oxalate, and some other specific toxic compounds, is frequent in some edible species when collected in the wild, so a moderate use is suggested. For example, nitrites bind to hamoglobin and reduce the transport of oxygen to tissues [43]. Furthermore, the capacity of nitrites to combine with amines produces nitrosamines, which are carcinogenic substances [43]. Oxalic acid can reduce the availability of calcium through the formation of an insoluble complex of calcium oxalate, known as raphide, which is the primary cause of the most common kind of kidney stones [74]. Thus, the development of species-specific cultivation protocols can be useful to limit the accumulation of possible toxic compounds in the species that are well appreciated by consumers.

*B. officinalis*, one of the most commonly eaten wild plants, should be consumed with precaution as it contains considerable amounts of hepatotoxic pyrrolizidine-based alkaloids, such as thesinine, lycopsamine, and intermedine, which are mildly mutagenic. Acute poisoning by pyrrolizidine alkaloids causes haemorrhagic necrosis, hepatomegaly, and ascites. The subacute toxicity is characterized by occlusion of the hepatic veins and subsequent necrosis, fibrosis, and liver cirrhosis [74]. Another wild species, *F. vulgare*, contains two toxic phenylpropanoids: estragole with hepatocarcinogenic activity; and *trans*-anethole, having genotoxic and hepatocarcinogenic properties [75].

The concentration of oxalate, nitrates, and other toxic compounds found in the selected wild edible species is given in Table 3.

## 4. Exploiting the Possibilities of Cultivation of Some Wild Mediterranean Edible Species: Preliminary Results, Perspectives and Opportunities

The Food and Agriculture Organization defines wild edible plants as: “Plants that grow spontaneously in self-maintaining populations in natural or semi-natural ecosystems and can exist independently of direct human action” [80]. However, the gap between the increasing human population and food availability is constantly enlarging, which requires protecting some plant species from imprudent harvesting. In addition, considering food safety, the phytochemical properties of food are a hot topic, especially in Western countries [80]. Therefore, it seems important to find an efficient cultivation method for wild species (though this contrasts with the definition of “wild species”) to allow a large-scale, high-yield production with a reproducible phytochemical profile, and in parallel, reduce the risks related to the presence of toxic compounds. Below we report some preliminary results from germination tests of some wild species (Table 4); and the biomass yield of *R. acetosa* and *S. minor* (Table 5), the two species that have demonstrated good potential for cultivation in a hydroponic system, an agronomic technique that ensures high yield and standardization in phytochemical profiles.

### 4.1. Germination Test

Usually, wild species collected in the wild are characterized by a reduced germination rate when compared to species that are commonly cultivated. In Table 4 we report the germination test of some potentially-interesting wild Mediterranean edible species, namely *P. oleracea*, *R. acetosa*, *S. vulgaris*, *S. minor*, *T. officinale*, and *U. dioica*. The germination rate was evaluated in Petri dishes in both dark and light (about 250–300 µmol quanta m^−2^ s^−1^) conditions at 27 °C and saturated relative humidity (25 seeds per Petri dishes; *n* = 3). The germination rate was calculated as the percentage of seeds germinated after ten days (Table 4). Within ten days, mean germination time was calculated as the mean of the days necessary to obtain the maximum germination (Table 4). The germination rate was found to be highly variable under different conditions, for example, under light conditions germination was very low in *U. dioica*, medium in *T. officinale* and *P. oleracea*, and very high in *S. vulgaris*, *R. acetosa*, and *S. minor* (the latter was similar to that of commercial seeds of *Eruca sativa* (L.) Mill.). We did not observe differences between the germination rate under dark or light conditions (*p* > 0.05), except for *P. oleracea* and *T. officinale*, for which the rate was significantly reduced in dark conditions (Student’s *t* test; *p* < 0.01). The mean germination time in light conditions was the lowest in *P. oleracea*, followed by *R. acetosa*, *S. minor*, and *T. officinale*, whilst *U. dioica* showed the highest. No remarkable differences were found among the species when the mean germination time of seeds grown under light was compared to that observed in dark conditions (*p* > 0.05).

### 4.2. The Cultivation 

**Figure 1:**
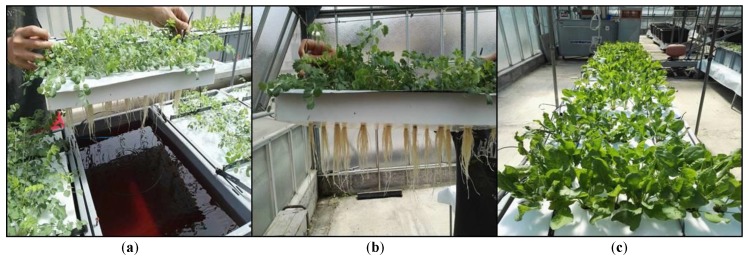
Floating system cultivation of *Sanguisorba minor* (**a**,**b**) and *Rumex acetosa* (**c**).

In addition to the low germination rate observed for some wild species, another critical point to overcome for the first stages of “domestication” of wild species is the establishment of a proper cultivation method. In many cases, wild species typically inhabit limiting environments, and are often slow-growing with very low biomass yield. The selection of the most promising genotypes can overcome this problem if implemented in association with the best cultivation practice that maximizes the biomass yield. Therefore, we utilized the hydroponic cultivation system (the floating system, Figure 1) given that it delivers better plant yields than soil culture, with less water usage and higher fertilizer efficiency. Some other authors [3] have indeed utilized the hydroponic system for the cultivation of wild medicinal plants, not only to maximize the plant yield, but even to selectively stimulate the biosynthesis of targeted metabolites, and/or to standardize the biochemical profile of these species [82]. Another important aspect that can be overcome with the utilization of a hydroponic system is the reduction of toxic compounds [83].

Taking into consideration the highest percentage of germination of *R. acetosa* and *S. minor*, these species were tested for their potential of cultivation in a floating system. Therefore, a pilot experiment was conducted in which these two species were grown under greenhouse conditions with natural light during the period April–June 2017 in the facilities of the Department of Agriculture, Food and Environment (University of Pisa, Pisa, Italy). The plants were hydroponically grown in a nutrient solution having the following composition: NO_3_^−^ 10 mM, NH_4_^+^ 0.5 mM, PO_4_^3−^ 1 mM, K^+^ 6 mM, Ca^2+^ 4 mM, Mg^2+^ 2 mM, Na^+^ 0.5 mM, SO_4_^2−^ 3.5 mM, Fe^2+^ 40 µM, BO_3_^−^ 25 µM, Cu^2+^ 1 µM, Zn^2+^ 5 µM, Mn^2+^ 10 µM, Mo^3+^ 1 µM. Electrical conductivity was 1.98 dS m^−1^ and pH values were adjusted to 5.7–6 with diluted sulphuric acid. The solution was kept continuously aerated, and replaced every week with a fresh one. 

Preliminary results concerning the cultivation of *R. acetosa* and *S. minor* in the floating system showed a lower yield than that of some commercial species (Table 5). However, with an appropriate manipulation of the nutrient solution, growing condition, and genotype selection, the challenge to increase the biomass yield of these species can realistically be addressed. However, in this study only very preliminary results are given, and to make a complete picture of the performance of these two species further investigations are needed. In addition, similar experiments need to be carried out with other wild edible species interesting as a source of healthy bioactive compounds, and the organoleptic characteristics of these species also need to be evaluated, as they are an important aspect for consumers.

### 4.3. Perspective and Opportunities for Wild Edible Species Cultivation

Ethnobotanical surveys show that more than 7000 species of wild plants have been used for human food at some point throughout human history, and that edible species are a regular component of the diets of millions of people [86]. Recent studies also pointed out that many people worldwide still rely on local environmental resources, especially wild plants, for daily subsistence and healthcare [87,88,89,90]. In different regions lacking basic infrastructure and market access, wild gathering provides considerable subsistence support to local diets [91], and may also generate further benefits (e.g., selling surpluses) [92]. However, in some cases gathering from the wild, and family farming and/or smallholder agriculture, are not enough to meet nutritional needs in developing regions [93], as was expressed in a report on the state of food insecurity in the world [94], which states, “progress towards food security and nutrition targets requires that food is available, accessible and of sufficient quantity and quality to ensure good nutritional outcomes”. Furthermore, in the near future, increasing human population, and continued globalization of trade and markets, along with ethnobotanical exploration, is expected to continue to increase awareness in the use of new plant materials. Therefore, the increase in demand for wild edible species will likely continue to threaten native species in some areas worldwide, as price differentials between wild and cultivated plants currently encourage unsustainable collection practices in some localities, especially in economically depressed regions that lack well-established rules for protecting wild plants [95]. 

Combining traditional knowledge and expertise with more recent concepts (e.g., public policies addressed to increasing human rights to food, health, and welfare, in addition to supporting plant biodiversity) is necessary for the benefit of future generations. The possibility to cultivate these wild edible species seems a promising approach to improve wild species yields and availability in a sustainable way, while protecting natural and crop biodiversity, as well as avoiding harmful anthropogenic contaminations of food, or the harvest of toxic species by inexperienced people. Research on the cultivation of wild species is in its infancy, and as also reported above, results indicate these species are still not competitive with more commercial species. However, there are significant possibilities to increase the yield of wild edible species has happened in the past for major crops, and this would encompass: (i) Selection of suitable species for their attitude to cultivation, (ii) breeding programs to selectively promote plant yield, and (iii) establishment of cultivation protocols to maximize plant performance. Of course, all these aspects should be considered in the context of local uses and economic possibilities; obviously the hydroponic technique represents just one of the possible cultivation techniques principally “affordable” in industrialized countries, whereas in other developing areas, other cultivation techniques have to be applied. In any case, cultivation will represent a step forward to: (i) Reduce the pressure of gathering in the wild, (ii) reduce the risk of food contamination, and (iii) diversify human diet and promote access to bioactive food. In this perspective, new ideas about food and health are welcome to respond to demand offood supply, quality, and safety.

## 5. Conclusions

Wild edible plants are widely present in the Mediterranean basin, and ethnobotany reports their cooking and medicinal use over a long time. Today, more than one billion people in the world utilize wild vegetables in their daily diet, especially in developing countries. Conversely, people of industrialized countries are “rediscovering” wild edible species for culinary use, as these wild vegetables add a variety of color, taste, and texture in their diet. It seems necessary to develop an efficient large-scale cultivation method for these species in order to standardize their yield and nutraceutical values. Nevertheless, in most cases, wild species can be toxic due to the high content of oxalic acid, nitrates, and sometimes, other toxic compounds [74]. Consequently, excessive consumption can cause some problems to human health, especially in infants [14]. Therefore, cultivation techniques can also be beneficial in controlling and limiting the accumulation of nitrates and oxalic acid. It is conceivable that with appropriate research addressed to improving these features, and with proper promotional marketing, these wild edible species may open up new commercial opportunities in the countries of the Mediterranean area. The nutritional and nutraceutical properties of these wild species make them especially charming considering the increasing attention amongst people towards the connection between food and health. In other words, some of these “neglected” species, sometimes considered as weeds in extensive major crop cultivation, may potentially become “new functional crops” in the not so distant future.

## Figures and Tables

**Table 1:** Bioactive phytoconstituent profile of fifteen Mediterranean wild species selected for their aptitude in cultivation.

Species	Family	Plant Part	Bioactive Phytoconstituents	Properties	References
*Allium ampeloprasum*	Liliaceae	bulbs, leaves	specific saponins (ampelosides Bs1, -Bf1, -Bf2, prosapogenin of aginoside, agigenin 3-*O*-β-glucopyranosyl(1→3)-β-glucopyranosyl(1→4)-β-galactopyranoside, (25R)-26-*O*-β-glucopyranosyl-22-hydroxy-5α-furostane-2α,3β,6β, 26-tetraol-3-*O*-β-glucopyranosil-22-hydroxy-5α-furostane-2α,3β,6β, 26-tetraol-3-*O*-β-glucopyranosyl(1→4)-β-galactopyranoside), allin, alliicin, γ-glutamyl peptides, S-alk(en)yl-l-ysteine sulphoxides (isoalliin, methiin, cycloalliin) α-limonene, β-pinene, 9-octadecanoic acid, hexadecanoic acid, *trans*-caryophylene, dimethyl-trisulfid, caryophylene oxide, phenolic acids, flavonoids, tannins	antifungal and antibacterial, antioxidant, hypoglycemic and hypolipidemic, against gastrointestinal disorders	[8,9,10,11,12]
*Asparagus acutifolius* L.	Asparagaceae	shoots	flavonoids, phenolic acids (caffeic acid, kaempferol, catechol, quercetin, isorhamnetin), carotenoids (lutein, β-carotene, neoxanthin, violaxanthin), steroidal saponins	radical scavenging and antioxidant, diuretic	[13,14,15,16]
*Borago officinalis* L.	Boraginaceae	leaves, shoots and roots	mucilage, tannins, saponins, flavonoids allantoin, rosmarinic acid, vitamin C, vitamin B1-B2-B3	antioxidant and pharmacological	[17,18,19,20]
*Cichorium intybus* L.	Asteraceae	leaves	flavonoids, terpenoids, carotenoids, hydroxicinnamic acids (HCA1-HCA2-HCA3-HCA4-HCA5-HCA6-HCA7-HCA8-HCA9-HCA10-HCA11), caffeic acid, caftaric acid, benzoic acid derivate (BAD), chlorogenic acid, some gallic acid derivatives (GAD1-GAD2), flavonols, anthocyanin, some unknown phenolic compounds, coumarins, sesquiterpene lactones, lactucin, lactucopicrin, α-linolenic acid, apigenin, astragalin, betain, tannins, cichoriin, inulin, kaempferol, quercetin, rutin, taraxasterol, vanillic acid, 2 new coumarin glycoside esters (cichoriin-69-*p*-hydroxyphenylacetate and benzyl-β-glucopyranoside)	antioxidant, antimalarial, digestive, anticancer	[21,22,23,24]
*Diplotaxis tenuifolia* (L.) DC.	Brassicaceae	leaves	flavonoids, polyphenols, glucosinolates (desulphoglucosinolates, pentylglucosinolate), glucoraphanin, glucoerucin, diglucothiobeinin, glucosativin, allyl sulphyde, sinapine, diplotaxilene, butylene	antioxidant, anticancer	[25,26,27,28]
*Foeniculum vulgare* Mill.	Apiaceae	shoots, leaves, stem, inflorescences	21 fatty acids (caproic acid, undecanoic acid, myristic acid, myristicoleic acid, capric acid, caprylic acid, lauric acid, pentadecanoic acid, heptadecanoic acid, oleic, linoleic and α-linoleic acid, stearic acid, eicosanoic acid, *cis*-11,14-eicosadienoic acid, arachidic acid, lignoceric acid), chlorogenic acid, reochlorogenic acid, gallic acid, caffeic acid, ferulic acid-7-*O*-glucoside, *p*-cumaric acid, quercetin-7-*O*-glucoside, dicaffeoylquinic acid, ferulic acid-7-*O*-glucoside, hesperidin, cinnamic acid, rosmarinic acid, quercetin, apigenin, eriodictyol-7-rutinoside, limonene-10-ol, isorhamnetin-3-*O*-glucoside, *cis*-miyabenol, dillapional, exo-fenchyl acetate, quercetin-3-glucoronide, quercetin-3-arabinoside, isoquercetin, kaempferol-3-arabinoside, isoquercetin, kaempferol-3-arabinoside, isorhamnetin glucoside, 3,4-dihydroxyphethylalchohol-6-*O*-caffeoyl-β-d-glucopyranoside, 3’,8’-binaringenin	antioxidant, hepatic activity, sebum-reducing agent, antimicrobial	[29,30,31,32,33,34]
*Malva sylvestris* L.	Malvaceae	flowers	anthocyanins (malvidin), vitamin C, alkaloids, saponins, flavonoids, tannins, phenolic compounds	reduction of coronary heart disease, antioxidant, anticancer, improved visual acuity	[20,24]
*Papaver rhoeas* L.	Papaveraceae	leaves, flowers	vitamin C, α-tocopherols, fumaric acid, citric acid, malic acid, tannins flavonoids	measles treatment, anti-nervousness, anti-insomnia, digestive, against respiratory disorders, anti-baldness, against eye infection	[2,35,36]
*Portulaca oleracea* L.	Portulacaceae	leaves, stems, roots, seeds	carotenoids, vitamin C, α-tocopherols, specific alkaloids (5-hydroxy-a-*p*-coumaricacyl-2,3-dihydro-1H-indole-2-carboxylicacid-6-*O*-β-d-glucopyranoside; 5-hydroxy-1-ferulicacyl-2,3-dihydro-1H-indole-2-carboxylic acid-6-*O*-β-d-glucopyranoside; 5-hydroxy-1-(*p*-coumaric acyl-7’-*O*-β-d-glucopyranose)-2,3-dihydro-1H-indole-2-carboxylicacid-6-*O*-β-d-glucopyranoside; 5-hydroxy-1-(ferulicacyl-7’-*O*-β-d-glucopyranose)-2,3-dihydro-1H-indole-2-carboxylicacid-6-*O*-β-d-glucopyranoside; 8,9-dihydroxy-1,5,6,10b-tetrahydro-2H-pirrolo[2,1-a]isoquinolin-3-one; oleracein A–E; (3R)-3,5-bis(3-methoxy-4-hydroxyphenyl)-2,3-dihydro-2(1H)-pyridinone and 1,5-dimetyl-6-phenyl-1,2-dihydro-1,2,4-triazin3(2H)-one), Oleracone, Oleracin I, Oleracin II (novel alkaloids), other alkaloids (trollisine, aurantiamide acetate, aurantiamide, scopoletin, dopamine, noradrenaline, N-*trans*.feruloyltyramine), saponines, phenolic acids (3-caffeoylquinic acid, 5-caffeoylquinic acid), coumarins, flavonoids (kaempferol, apigenin, luteolin, myricetin, quercetin), 4 homoisoflavonoids (portulacanones A–D), tannins, terpenoids (Portuloside A-B, portulene, lupeol; (3S)-3-*O*-(β-d-glucopyranosil-3,7-dimethylocta-1,6-dien-3-ol; (3S)-3-*O*-(β-d-glucopyranosil)-3,7-dimethylocta-1,5-dien-3,7-diol; (2α,3α)-3-{[4-*O*-(β-d-glucopyranosyl)- β-d-xylopyranosyl}-2,23-dihydroxy-30-methoxy-30-oxoolean-12-en-28-oic acid; (2α,3α)-2,23,30-trihydroxy-3-[β-d-xylopyranosil)oxy]olean-12-en-28-oic acid; friedelane), organic acids (α-linolenic acid, palmitic acid, linolenic acid), portulacerebroside A, melatonin	food coloring agents, antioxidant and radical scavenging, anti-inflammatory, analgesic, antifungal, antibacterial, antiscorbutic, depurative, diuretic and febrifuge. Fresh juice is used in the treatment of strangury, coughs, sores. Both leaves and plant juice are effective in the treatment of skin diseases and insect stings. The infusion of leaves is used against stomach aches and headaches	[37,38,39,40,41,42,43,44]
*Rumex acetosa* L.	Polygonaceae	leaves and shoots	6-methyl-1,3,8-trichlorodibenzofuran, chrysophanol, physcion/parietin, emodin-8-*O*-β-d-glucopiranoside, naphthalene-1,8-diol, catechin/epicatechin, epicatechina-3-*O*-gallate, vitexine, vanillic acid, sinapic acid, procyanidin B2 3'-*O*-gallate, pulmatin, gallocatechin/epigallocatechin, procianidin B2, geraniin, corilagin, ellagic acid, rosmarinic acid, pyrogallol	anti-mutagenic and anti-proliferative activities	[45,46,47,48]
*Sanguisorba minor* Scop.	Rosaceae	leaves	linalool, nonanal, dodecane, tridecane, α-damascenone, tetradecane, β-caryophyllene, hexadecane, heptadecane, octadecane, (E-E)-farnesyl acetate, nonadecane, eicosane, heneicosane, docosane, β-sitosterol, caffeic acid, kaempferol, quercetin	digestive properties, antioxidant, astringency, carminative, diuretic	[16,49,50,51,52]
*Silene vulgaris* (Moench) Garcke	Caryophyllaceae	leaves	linoleinc and α-linolenic acids, vitamin C, silenan SV, vitamin E, quinic acid, malic acid, *trans*-aconitic acid, chlorogenic acid, protocatechuic acid, *p*-coumaric acid, hesperidin, rutin, hyperoside	antifungal, anti-enzymatic, antimicrobial and antioxidant, immunomodulatory	[7,53,54]
*Sinapis arvensis* L.	Brassicaceae	essential oils, flowers and leaves	monoterpenes, sesquiterpenes, nitriles aldehydes, sulphur-containing compounds benzylisothiocyanate, cubenol, dimethyltrisulfide, 6,10,14-trimethylpentadecane-2-one, indole, 1-butenylisoithiocyanate, thymol, octadecane, spathulenal, hexadecane, 1-epi-cubenol, octadecanol 2-phenilisothiocyanate, δ-cadinene, 9-methylthiononanonitrile, nonadecane, octadecanal, flavonoids (low amount), alkaloids, saponins	tonic, diuretic, expectorant, febrifuge, stomachic, antiscorbutic, antioxidant, spices	[55,56]
*Taraxacum officinale* Web.	Asteraceae	flowers, roots, stems and leaves	tetrahydroridentine B7, taraxacolide-1-*O*-β-d-glucopyranoside, taraxeryl acetate/taraxerol acetate, taraxic acid, taraxacoside, taraxasterin/taraxasterol/taraxol/β-amirin, taraxafolide, 4,13,11,15-tetrahydroredentine, 11β,13-di-hydrolattucine, ixerin D, arnidiol/faradiol, dihydroconiferine, sitosterol, stigmasterol, apigenin-7-glucoside, luteolin-7-glucoside, luteolin 7-*O*-rutinoside, quercetin 7-*O*-glucoside, taraxastane carotenoids, saponins, alkaloids, flavonoids 4 anthocyanins: cyanidin-3-glucoside, cyanidin-3-(6-malonyl)-glucoside A-1; cyanidin-3-(6-malonyl)-glucoside A-2), peonidin-3-(malonyl) glucoside, cicoric acid, sinapic acid, caffeic acid, ferulic acid, *p*-hydroxyphenylacetic acid, chlorogenic acid, *p*-cumaric acid	analgesic, antirheumatic, cholagogue, diuretic, laxative, hypocholesterole eupeptic, digestive, antioxidant	[16,57,58,59,60,61]
*Urtica dioica* L.	Urticaceae	leaves and young sprouts	carotenoids (lutein and β-carotene), anthocyanins, hydroxycinnamic acid derivates (chlorogenic acid, dihydrosinapoyl alcohol) vitamin C, flavonoids, lignans	antioxidant, against stomach ache, against rheumatic pain, against colds and cough, against liver insufficiency and hypertensive, anti-inflammatory and diuretic	[62,63,64,65]

**Table 2:** Traditional recipes prepared with the fifteen Mediterranean wild edible species that have been selected in this review for their aptitude for cultivation.

Species.	Edible Part	Traditional Recipes	References
*Allium ampeloprasum*	leaves and bulbs	mixture of salads, omelet, boiled vegetables, soup	[66]
*Asparagus acutifolius*	young shoots	boiled with oil and vinegar, omelet, risotto, soup	[66]
*Borago officinalis*	tender rosette	boiled with olive oil, salt, lemon and vinegar; stewed, omelet, soup, home-made pie	[67,68,69]
*Cichorium intybus*	tender leaves	fresh salads, in pan with olive oil and garlic, pies, ravioli, soup	[66]
*Diplotaxis tenuifolia*	fresh leaves	mixed salads, pies, pasta, omelet, cheeses, pizza	[70]
*Foeniculum vulgare*	fruits, seeds, leaves	salads, snacks, boiled, grilled, stewed vegetables, bread, soup	[29,66]
*Malva sylvestris*	fresh leaves	ravioli, omelet, meatball, soup	[66]
*Papaver rhoeas*	basal rosette leaves	salads, ravioli, bread, soup	[66]
*Portulaca oleracea*	leaves	salads	[71]
*Rumex acetosa*	young leaves, stems	salads, fried, sautéed with butter and lard, pies, raw snacks	[72]
*Sanguisorba minor*	young leaves	salads, boiled vegetables, soup and pureed soup	[66]
*Silene vulgaris*	old leaves	salads, boiled, fried, sautéed with garlic, omelet	[73]
*Sinapis arvensis*	leaves	spice as mustard	[67]
*Taraxacum officinale*	basal leaves	salad, in pan with olive oil and garlic, ravioli, soup, pie	[66]
*Urtica dioica*	leaves, young sprouts	risotto, pie, ravioli, boiled, cooked in pan with olive oil and lemon, omelet, soup and pasta	[66]

**Table 3:** Concentration of toxic compounds in some Mediterranean wild edible species.

Species	Toxic Compounds	Concentration	References
*Allium ampeloprasum*.	oxalic acid	11.13 ± 0.48 and 6.32 ± 0.65 mg/100·g (two different populations)	[76]
*Borago officinalis*	pyrrolizidine alkaloid: amabiline, thesinine, intermedine, and lycopsamine	n.d. ^3^	[52,74]
*Cichorium intybus*	nitrate	75 mg kg^−1^ FW ^1^	[77]
	oxalic acid	8.68 ± 0.05 and 3.00 ± 0.71 mg/100 g (two different populations)	[76]
*Diplotaxis tenuifolia*	nitrate	3874 mg kg^−1^ FW ^1^	[77]
*Foeniculum vulgare*	phenylpropanoids: *trans*-anethole and estragole	2.3–4.9% (aerial parts)	[74]
	phenylpropanoid: estragole	0.8 – > 80%	[74]
	phenylpropanoid: estragole	from 11.9 to 56.1% in unripe seeds to 61.8% in ripe seed	[74]
	oxalic acid	123.82 ± 8.75 and 402.83 ± 21.87 mg/100 g (two different populations)	[76]
*Papaver rhoeas*	nitrate	>2.500 mg·kg^−1^ FW ^1^	[70]
	oxalic acid	490.00 ± 27.05 and 428.65 ± 63.63 mg/100 g (two different populations)	[76]
*Portulaca oleracea*	nitrate	48.98 (leaf) and 43.90 mg g^−1^ (steam) DW ^2^	[78]
	oxalic acid	1.27 (leaf) and 0.55 mg g^−1^ (steam) DW ^2^	[78]
*Rumex acetosa*	oxalates and hydroxyanthracene derivatives: chrysophanol, physcion, emodin, aloe-emodin, rhein, barbaloin (aloin A and B), and sennosides A and B	n.d. ^3^	[74]
*Silene vulgaris*	triterpenoid saponins	n.d. ^3^	[74]
	silenosides A, B	n.d.^3^	[76]
	and C oxalic acid	201.79 ± 15.98 and 218.73 ± 17.56 mg/100 g (two different populations)	
*Sinapis arvensis*	nitrate	3028 mg kg^−1^ FW ^1^	[77]
*Taraxacum officinale*	sesquiterpene lactone taraxinic acid β-glucopyranosyl ester	n.d. ^3^ .	[61]
*Urtica dioica*	nitrate	849–1631 mg kg^−1^ FW ^1^	[79]

^1^ FW: fresh weight; ^2^ DW: dry weight; ^3^ n.d.: not determined

**Table 4:** Percentage of germination and mean germination time of seeds of *Portulaca oleracea, Rumex acetosa, Sanguisorba minor, Silena vulgaris, Taraxacum officinale*, and *Urtica dioica* in light and dark conditions. Means were compared by one-way analysis of variance with species as the variability factor. Means keyed with different letters (in the same column) are significantly different following Fisher’s least significant difference post-hoc (*p* = 0.05). Percentage values were arcsine transformed prior analyses.

	Germination (%)		Mean Germination Time (Days)	
Species	Light	Dark	Light	Dark
*Portulaca oleracea*	64 ± 8 c	51 ± 2 c	3.3 ± 0.3 d	3.7 ± 0.7 bc
*Rumex acetosa*	96 ± 4 a	92 ± 1 a	3.5 ± 0.4 cd	3.5 ± 0.2 c
*Sanguisorba minor*	97 ± 5 a	99 ± 2 a	3.7 ± 0.2 cd	3.9 ± 0.3 bc
*Silene vulgaris*	79 ± 6 ab	76 ± 8 b	5.3 ± 0.6 b	5.0 ± 0.8 b
*Taraxacum officinale*	59 ± 5 c	45 ± 6 c	4.3 ± 0.4 c	4.4 ± 0.6 bc
*Urtica dioica*	11 ± 2 d	9 ± 6 d	7.8 ± 1.0 a	8.5 ± 1.5 a
*Eruca sativa* (data of [81])	88 ± 6 a	n.d.^1^	n.d.^1^	n.d. ^1^

^1^ n.d.: not determined.

**Table 5:** Biomass yield of hydroponically-cultivated *Rumex acetosa* and *Sanguisorba minor, Valerianella locusta* L. Laterr., and *Eruca sativa.* Data are the mean (± SD) of three independent replicates.

Plant Species	Biomass Yield (g Fresh Weight m^−2^ day^−1^)
*Rumex acetosa*	29.5 ± 1.8
*Sanguisorba minor*	22.7 ± 1.3
*Valerianella locusta* [84]	38 ± 2.0
*Eruca sativa* [85]	67.5 ± 5.8
